# Modelling of Fatigue Microfracture in Porous Sintered Steel Using a Phase-Field Method

**DOI:** 10.3390/ma16114174

**Published:** 2023-06-03

**Authors:** Zoran Tomić, Tomislav Jarak, Tomislav Lesičar, Nenad Gubeljak, Zdenko Tonković

**Affiliations:** 1 Faculty of Mechanical Engineering and Naval Architecture, University of Zagreb, 10000 Zagreb, Croatia; zoran.tomic@fsb.hr (Z.T.); tomislav.lesicar@fsb.hr (T.L.); zdenko.tonkovic@fsb.hr (Z.T.); 2 ITAP, School of Industrial Engineering, University of Valladolid, Paseo de Cauce 59, 47011 Valladolid, Spain; 3 Faculty of Mechanical Engineering, University of Maribor, 2000 Maribor, Slovenia; nenad.gubeljak@um.si

**Keywords:** sintered steel, microcracks, fatigue, phase-field method, porosity

## Abstract

Porosity in sintered materials negatively affects its fatigue properties. In investigating its influence, the application of numerical simulations reduces experimental testing, but they are computationally very expensive. In this work, the application of a relatively simple numerical phase-field (PF) model for fatigue fracture is proposed for estimation of the fatigue life of sintered steels by analysis of microcrack evolution. A model for brittle fracture and a new cycle skipping algorithm are used to reduce computational costs. A multiphase sintered steel, consisting of bainite and ferrite, is examined. Detailed finite element models of the microstructure are generated from high-resolution metallography images. Microstructural elastic material parameters are obtained using instrumented indentation, while fracture model parameters are estimated from experimental S–N curves. Numerical results obtained for monotonous and fatigue fracture are compared with data from experimental measurements. The proposed methodology is able to capture some important fracture phenomena in the considered material, such as the initiation of the first damage in the microstructure, the forming of larger cracks at the macroscopic level, and the total life in a high cycle fatigue regime. However, due to the adopted simplifications, the model is not suitable for predicting accurate and realistic crack patterns of microcracks.

## 1. Introduction

In recent years, much effort has been made in investigating the influence of material microstructure on the failure mechanisms of highly heterogeneous materials, such as sintered materials, using various numerical and experimental techniques. This is especially important when they are subjected to cyclic loading conditions. Structural components manufactured with various sintering or additive technologies are becoming attractive due to their good vibration and noise reduction properties, accompanied by a very high rate of raw material utilization in such processes [1]. This is especially favorable for large-series production of machine components with complex geometry, such as gears, spurs or bearings. However, pores appear in sintered materials, which are often a predominant factor in failure mechanisms in dynamically loaded sintered parts [2,3]. Microcracks usually initiate around smaller closely packed pores, propagate to nearby pores and eventually mutually coalesce, creating a macrocrack which can finally cause the failure of a component. Being able to predict the relationship between material microstructure and fracture behaviour by means of numerical modelling could significantly reduce the amount of time and costs needed for identifying the “optimal” microstructure.

Most often, models based on the Finite Element Method (FEM) have been used for investigating material microfracture, due to the robustness and availability of FEM. The Extended Finite Element Method (XFEM), based on enriching the approximation functions space with discontinuous functions, has sometimes been used; see, e.g., [4,5]. Nevertheless, in XFEM it is difficult to accurately track crack geometries in problems where multiple cracks evolve, branch or merge. Cohesive Zone Modelling (CZM) has sometimes been viewed as a good alternative to XFEM, see, e.g., [6,7], but to predict the paths of arbitrary cracks, cohesive elements have to be used along all finite element edges [8], which is computationally expensive. Further interesting models to be considered for understanding microfracture might be models with embedded and smeared discontinuities [9]. In problems with arbitrary crack paths, remeshing techniques are often used, but they require special numerical procedures and special attention has to be dedicated to avoiding loss of accuracy during mapping. Recently, the moving mesh method [10] has been proposed to tackle this problem, where the Arbitrary Lagrangian formulation (ALE) is used to simulate the evolution of material discontinuities.

During the last decade, phase-field (PF) modelling of fracture experienced significant popularity, since no ad hoc criteria are needed for crack initiation or propagation, and complex crack patterns can be modelled without the need to explicitly track crack geometries. Therefore, such PF methods seem to be appropriate for modelling cracking in the microstructures of heterogeneous materials. Thereby, some important issues are the modelling of cracking along material interfaces or crystal grains and model calibration.

Simple multiphase PF models for fracture, where the effects of interfacing between the particles or grains or anisotropy are not modelled, have been considered in the literature for reproducing global material responses. In [11] the topology of a highly heterogeneous realistic microstructure was extracted from X-ray CT images, and the improved formulation proposed by Miehe et al. [12] was used to analyse fracture. A similar formulation was used for 3D analysis [13] using models created from microtomography. To cut computational costs, Seleš et al. [14] proposed a staggered solution procedure based on a residual norm with improved convergence properties to study size effects associated with microscale fracture in a hypothetical material.

More sophisticated models have been developed to include the anisotropy of grains or to describe intergranular cracks [15,16,17,18,19,20,21,22,23]. The grain boundaries are often modelled as smeared internal layers between grains with fracture toughness dependent on the surrounding bulk material [15,17], or as an additional material phase appearing as a thin layer between grains [19,20]. Alternatively, a Cohesive Zone Model (CZM) might be used to model intergranular cracks [21,22]. Various models with multiple phase field parameters may also be found in the literature [17,18,23]. Common issues in such sophisticated models are the mechanical characterization of the boundaries and the increased complexity. They also demand a higher computational burden, which is in general one of the main obstacles in the practical use of PF models. This could become even more pronounced in fatigue computations for porous sintered materials, where complex geometries require the application of dense finite element meshes everywhere in the model to accurately capture geometry and phase-field evolution. The recent work by the authors of [24] represents an early attempt at fatigue microfracture analysis, where a fatigue fracture in nodular cast iron was simulated using a generalized PF model [25], featuring friction contact between elastic graphite nodules and the elasto-plastic metallic matrix. However, in that work, validation results for total life estimation were not offered, and the calibration of such a model is still an open question.

In general, one of the main problems in numerical material modelling at the microstructural level is the mechanical characterization of material phases. Using different indenters and different forces, it is possible to characterize single grains of material from nano- to macroscale. The basic method was proposed by Oliver and Pharr [26], which enables reliable estimation of elastic properties. Later, various methods for extracting complete stress–strain curves of individual grains were presented [27,28,29]. Recently, instrumented indentation has been applied for the mechanical characterization of plastic parameters in a multi-phase steel [30], but the reliability and accuracy of such data may be questionable, and from our experience depend significantly on the equipment used and the inverse method for characterization of plastic parameters.

This work deals with investigating the influence of porosity on fatigue failure in a sintered steel by employing PF modelling to observe the evolution of microcracks due to cyclic loading. A simplified PF model for brittle fatigue fracture with a threshold is applied. Fatigue is modelled by degrading the fracture toughness. It is derived from the general PF model presented in [24] by neglecting plastic deformation. Elastic local (microstructural) material properties are identified using instrumented indentation. In addition, the fracture toughness of individual phases is also estimated via indentation and used in the model as the initial fracture toughness (the fracture toughness of material undegraded by fatigue). The degradation of fracture toughness is modelled by a logarithmic function with one material fatigue parameter, which is calibrated from experimental S–N curves. Two-dimensional (2D) microscopic volume elements (MVEs) of different sizes are extracted from high-resolution metallography imaging and divided into spatial zones corresponding to the metallic phases observed in metallography images. In this work, it is assumed that the main driving feature for crack initiation and propagation through the microstructure in the considered material is porosity. The influence of grain boundaries and grain anisotropy is not accounted for in order to simplify the model and reduce the computational burden. The results of numerical simulations are compared with macroscale quasi-static uniaxial tension test (UTT) data and images obtained with a scanning electron microscope (SEM) to interpret the obtained results. Although material interfaces and plastic deformation could have significant impacts that cannot be captured by the present model, the obtained results indicate that the applied strategy is still capable of capturing some important phenomenological fatigue features, such as S–N curves.

The main highlights of the paper are:

- simplified numerical modelling based on the PF method for estimating the fatigue life of sintered steels is presented,
- mechanical characterization of the applied PF fatigue model based on the degradation of fracture energy is critically discussed,
- the influence of the porosity of the considered sintered steel on the estimated fatigue life is analysed using the proposed numerical method.

The paper is structured as follows: in Section 2 basic information about the applied numerical and experimental methods is given, including the governing equations of numerical phase-field formulations. The modelling process is also described. Afterwards, in Section 3 numerical results for monotonic quasi-static and dynamic fatigue fracture are presented, together with discussion of the results. Finally, some concluding remarks and future lines of research are proposed in Section 4.

## 2. Materials and Methods

### 2.1. Experimental Testing

**Figure 1:**
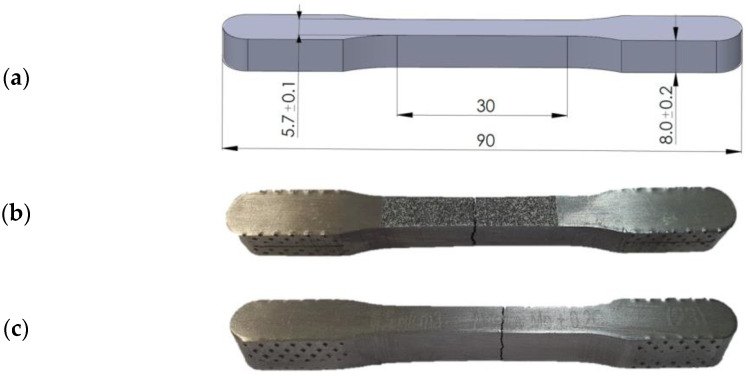
Sintered specimen according to ASTM E8: (**a**) dimensions, (**b**) fracture after quasi-static uniaxial tension test, (**c**) fatigue crack after cyclic uniaxial test.

For the purposes of this study, sintered samples (Figure 1) were compacted from Astaloy Mo+0.2C steel according to Höganäs [31]. Three densities were compacted: 6.5 g/cm^3^, 6.8 g/cm^3^ and 7.1 g/cm^3^, with dimensions according to the ASTM E8 standard [32]. More details about the compacting, sintering and cooling procedure can be found in the authors’ previous work [33]. The specimens in Figure 1 were used for the macroscopic uniaxial tension test (UTT), and smaller material samples were extracted from them for the purpose of metallographic and instrumental indentation testing.

#### 2.1.1. Microscale

**Figure 2:**
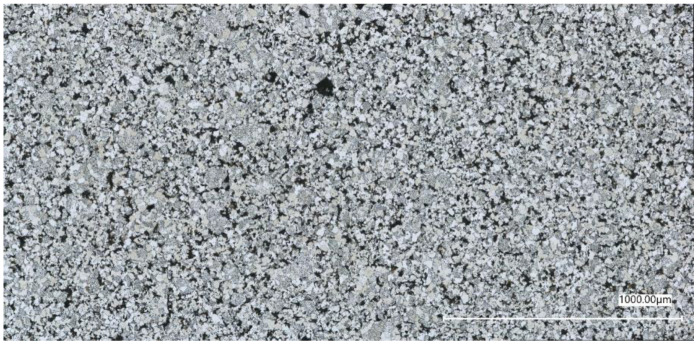
Typical microstructure of sintered Astaloy Mo+0.2C steel (here shown for specimen with 6.5 g/cm^3^). Metallography image shows a heterogeneous microstructure consisting of ferrite and bainite phases and pores (black color).

The microstructure consists of two distinct phases, ferrite and bainite, and is of relatively high porosity. Typical microstructure of the sintered steel is shown in Figure 2.

Table 1 shows some of the micromechanical properties for all three densities, obtained via instrumented Vickers indentation and high-resolution metallography imaging. The elastic modulus of different constituents was determined by the standard Oliver and Pharr method [26]. Indentation testing was conducted on an Anton Paar MCT^3^ testing device applying a standard diamond Vickers indenter. For more information about the indentation procedure, please see [33].

As reported in [33], the local (microscopic) values of the elastic properties for bainite and ferrite are almost identical for different densities, as shown in Table 1. This finding implies that the elastic macroscopic response of the considered material is driven mostly by porosity.

**Figure 3:**
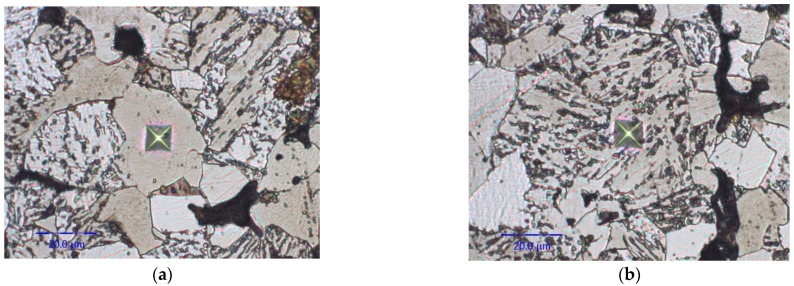
Typical residual Vickers indent in sintered steel: (**a**) ferrite phase, (**b**) bainite phase.

Besides the elastic properties of ferrite and bainite, the PF model for brittle fracture presented in Section 2.2 requires the material fracture toughness value. Although this parameter itself is not actually relevant for our analysis, here the fracture toughness of each material constituent was estimated via instrumental indentation and used to calculate the initial values of critical fracture energy (critical fracture energy before the onset of fatigue damage) in the model. At the moment there exist few works dealing with estimation of the fracture toughness of ductile materials using instrumented indentation; several methods have been proposed, such as those of Palmquist or Anstis [34,35] or Jeon et al. [36]. However, determination of the fracture toughness of ductile materials using indentation is a difficult task, because no cracks appear on the surface even when Berkovich or Vickers indenters are used; see Figure 3.

**Figure 4:**
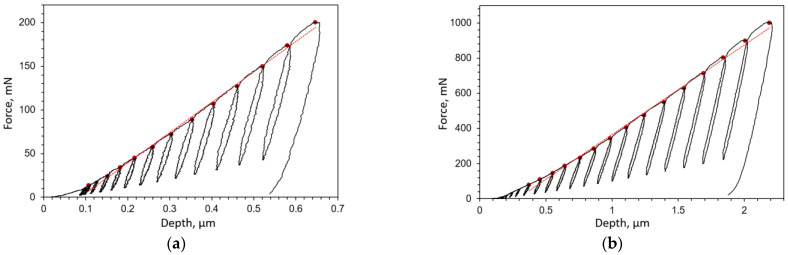
Force–displacement curves obtained via multicycle spherical indentation used for fracture toughness estimation of material constituents: (**a**) ferrite, (**b**) bainite.

The method used here was proposed by Jeon et al. [36]. It utilizes multicycle spherical indentation and is based on the theoretical background of plasticity evolution under an indenter proposed by Hertz. Multicycle indentation was used to obtain the required information, which was fed into an empirical equation. Indentation was conducted using a spherical indenter with a 50 µm radius and an Anton Paar Micro Combi Tester MCT^3^ at room temperature. At least 15 individual indentations were conducted for each phase. For the ferrite phase, the maximum force of 200 mN was achieved in 15 steps (Figure 4a), while for the bainite phase, the maximum force of 1000 mN was reached in 20 steps (Figure 4b). The load in each step was increased in an approximately linear fashion (see the red line in Figure 4). The sample surface was prepared according to the procedure described in [33]. Different maximum forces were chosen for testing each phase, with the maximum forces chosen so as to minimize the influence of phase interfaces and material surrounding the tested phase. However, since in this work bainite is considered an isotropic homogeneous material phase in numerical models, relatively high force was applied for bainite indentation to implicitly capture the influence of the heterogeneity of the bainite phase in the obtained results. The obtained fracture toughness values for bainite and ferrite are given in Table 2.

#### 2.1.2. Macroscale

**Figure 5:**
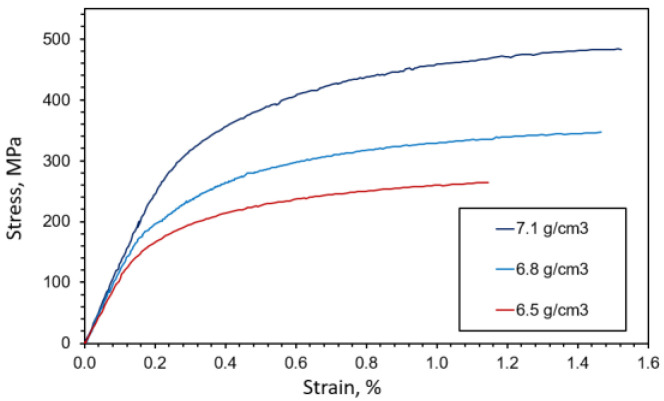
Mean stress–strain curves at a macroscopic level [35].

The sintered specimens were subjected to quasi-static tension loading in UTT. The obtained stress–strain curves, shown in Figure 5, serve in this article only for comparison with the results of numerical simulations of microfracture caused by quasi-static monotonous loading. More information about the static testing procedure undertaken can be found in [33].

As visible from Figure 5, a small plastic deformation is present at the macroscopic level before failure. In addition, elongation before failure decreases with increased porosity. This could be explained by the fact that in porous materials smaller particle necks between two adjacent pores promote higher localized plastic flows, which contribute to faster damage evolution at the microlevel and finally result in lower deformation of the loaded system, which is consistent with the observations in [2]. As is visible from metallographic imaging (Figure 2) and other presented figures from numerical models, in specimens with higher porosity there is a high probability that a larger number of small particle necks is encountered. This increases the number of probable locations for microcrack initiation, and a larger number of simultaneously propagating microcracks facilitates their coalescence and causes complete material failure before significant macroscopic plastic deformation occurs. Porosity also decreased the macroscopic yield stress, tensile strength and elastic modulus of the considered sintered steel. For all three considered microstructures, after the onset of fracture, a sudden failure producing a very sudden drop in stress was observed, typical for brittle fracture.

**Figure 6:**
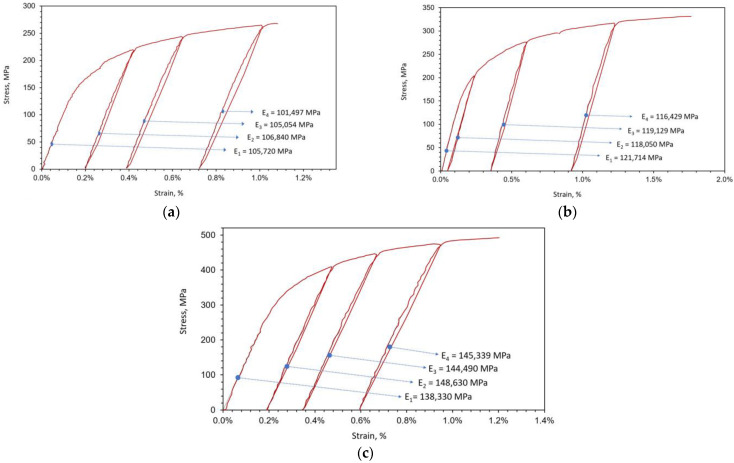
Uniaxial tension loading–unloading cycles for (**a**) 6.5 g/cm^3^, (**b**) 6.8 g/cm^3^, (**c**) 7.1 g/cm^3^.

Next, loading–unloading experiments were conducted with a Rumul Testronic 50 kN loading device, coupled with a GOM Aramis as a video-extensometer, making live tension–relaxation supervision possible. In the results of the loading–unloading tests (Figure 6), no change in elastic slope after unloading–loading cycles is visible (except for measurement error up to 2%). This means that microfracture events (if present) were not observable on the macroscale at stress levels lower than the ultimate tensile strength. This absence of damage at the macroscale before the occurrence of failure implies that plasticity could be a driving mechanism of failure in the considered material.

**Figure 7:**
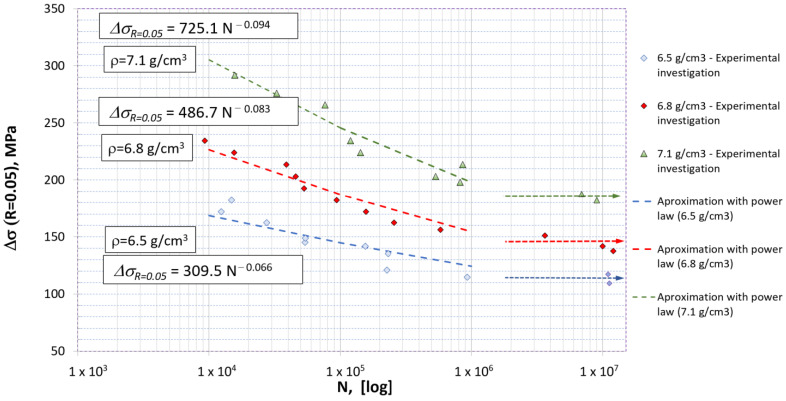
Wöhler curves for sintered steel specimens with 6.5, 6.8 and 7.1 g/cm^3^. The arrows pointing rightwards represent specimens that reached the fatigue limit, determined as more than 10^7^ cycles.

Fatigue tests were conducted on a Rumul 50 kN frequency resonant machine. The average testing frequency was around 130 Hz for High Cycle Fatigue (HCF). The surfaces of all sintered samples were previously polished using polishing paper of identical grade in order to maintain traceability. The loading ratio $R=F_{\max}/F_{\min}=\sigma_{\max}/\sigma_{\min}$ was maintained at 0.05. All tests were controlled by force. The Wöhler curves for all three densities are shown in Figure 7, together with the corresponding identified curve exponents and coefficients.

From Figure 7 it is visible that the total fatigue life significantly decreased with respect to increased porosity. For an identical loading cycle the 7.1 g/cm^3^ density steel might reach its fatigue endurance limit, while the 6.5 g/cm^3^ density steel might exhibit LCF behavior with only a few thousand cycles needed to reach the failure of the specimen.

#### 2.1.3. Fractographic Observations

**Figure 8:**
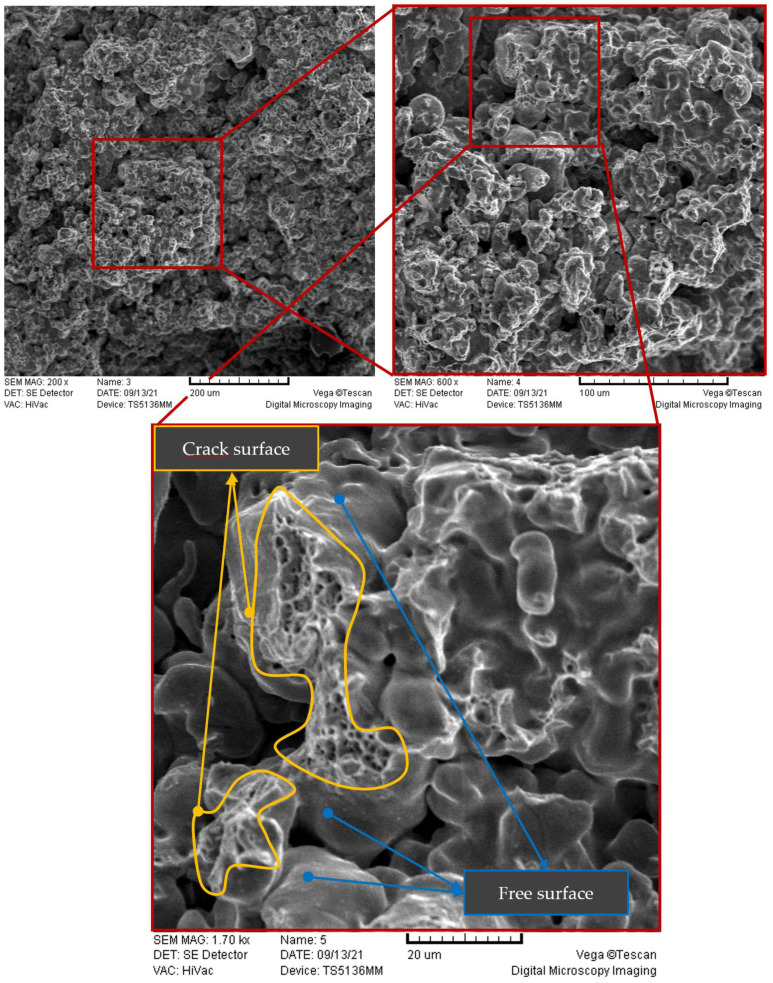
SEM imaging of the crack surface at different magnifications.

The results of the UTT suggest that plastic deformation should be the prominent process at the microscale as well, causing or contributing to the initiation of the first microcracks. Indeed, the crack surfaces observed in Scanning Electron Microscopy (SEM) images created after quasi-static uniaxial testing (Figure 8) were similar in all considered samples and correspond to the ductile dimple fracture mode. This is consistent with the findings from [3], where microfractographic analysis of Astaloy CRL + 0.7 wt-%C sintered steel was performed for both static and fatigue failure. In addition, similarly to the observations from [2,3,37], the microcrack patterns clearly show that a crack surface (marked by a yellow curve in Figure 8) is generally formed by a collapse of a ‘’bridge’’ between two pores (i.e., fracture of the neck joining two adjacent powder particles). The free surfaces marked in Figure 8 represent the walls of the surrounding pores. This also means that in such cases the microcrack is often formed as a transgranular crack (a crack passing though only one material phase), because metallographic analyses have shown that often only one material phase is present along the small bridge.

### 2.2. Numerical Phase-Field Model for Fatigue Fracture

The brittle PF model for fatigue fracture applied in this work is derived from the general plane strain PF formulation proposed in [25], omitting plastic deformation. The model was implemented in the commercial FE software Abaqus using UMAT and UEL user-defined functions.

In the following, only a brief overview is given, as more details can be found in [24,25]. Here, however, we will correct certain errors and clear out some ambiguities regarding this model that are encountered in previous works [24,25].

#### 2.2.1. Governing Equations

Starting from [38,39], in the general case of brittle fracture, the internal energy functional $\Psi^{\mathrm{int}}$ of a body Ω bounded by an outer surface ∂Ω and containing a crack with surface Γ can be defined as a sum of the strain energy in the bulk material $\Psi^{b}$ and the fracture energy $\Psi^{f}$ (energy dissipated by the fracture):

(1) $$\Psi^{\mathrm{int}}=\Psi^{b}+\Psi^{f}\approx \int_{\Omega /\Gamma }g\left(\phi \right)\psi_{e}\left(\varepsilon \right)\;dV+\int_{\Omega }\psi^{c}\left(\bar{\psi }\right)\;\left(2\phi +l\;{\left|\nabla \phi \right|}^{2}\right)dV$$

The scalar parameter ϕ is called the phase-field parameter and is used to define the intensity of damage in the material. It is a smooth scalar field that describes a transition from an intact material (ϕ=0) to a completely broken material (ϕ=1). Since fracture causes the loss of the initial stiffness of a material, strain energy is reduced by introducing a monotonically decreasing degradation function g(ϕ), with $\psi_{e}\left(\varepsilon \right)$ being the elastic strain energy density of the intact material (effective strain energy). In this work, the standard quadratic function $g\left(\phi \right)={\left(1-\phi \right)}^{2}$ is adopted. In passing, we note that in [24,25], the contribution of plastic deformation is included in the strain energy to model ductile fracture. To prevent unphysical crack propagation in compressive stress states, an appropriate strain energy decomposition should be introduced, which considerably increases the computational costs. However, in this work only problems with tension loading are studied, and it is deemed that in such cases, tensile stress states are predominant. Therefore, in Equation (1) an isotropic degradation of strain energy is employed, where the entire strain energy is degraded, and this issue of implementing an energy decomposition method is outside of the scope of this article.

The fracture energy $\Psi^{f}$ can be approximated as a domain integral according to Miehe et al. [39], where $\psi^{c}$ stands for a specific fracture energy. It can be shown that $\psi^{c}$ serves as the energetic threshold for fracture initiation in the material, and therefore such models are sometimes called PF models with thresholds (or threshold PF models). $\psi^{c}$ is the constant parameter in the case of quasi-static fracture caused by monotonous loading, but in fatigue formulations such as [38], it is assumed that fracture resistance degrades under the influence of repeated cycling load. This can be achieved by making $\psi^{c}$ dependent on the energy accumulation variable $\bar{\psi }$, which measures the “mileage” of the material subjected to repeated cycling loading by memorizing repeated strain energy changes during the loading history. Here $\bar{\psi }$ is calculated at a certain moment *t* of the loading process as

(2) $$\bar{\psi }\left(t\right)=\int_{0}^{t}{\dot{\psi }}_{e}\left(\tau \right)\;H\left({\dot{\psi }}_{e}\left(\tau \right)\right)d\tau$$

where *H* is the Heaviside step function, with a value of 0 when the strain energy in the material decreases (${\dot{\psi }}_{e}\leq 0$) and a value of 1 when the strain energy in the material increases (${\dot{\psi }}_{e}>0$) during the loading cycle. It is obvious that $\bar{\psi }$ has the character of a loading history(path-dependent) variable that increases only when strain energy grows during the loading process. It is of note that this definition of (2) is by no means the unique one. Finally, *l* denotes the length-scale parameter that controls the width of the transition zone between intact and completely broken material, where the damage intensity is described by the distribution of the phase-field ϕ.

**Remark.**  *In an attempt to quantify*$\psi^{c}$, *in* [24,25] *the fracture energy was represented in a manner typical for brittle PF models based on Griffith’s theory:*

(3) $$\Psi^{f}\approx \int_{\Omega }\psi^{c}\left(\bar{\psi }\right)\;\left(2\phi +l\;{\left|\nabla \phi \right|}^{2}\right)dV=\int_{\Omega }G_{c}\left(\bar{\psi }\right)\;\gamma \left(\phi ,\nabla \phi \right)dV$$

*where* γ(ϕ,∇ϕ) *is the surface crack density function, defined as*

(4) $$\gamma \left(\phi ,\nabla \phi \right)=\frac{3}{8\sqrt{2}}\left(\frac{2\phi }{l}+l^{2}{\left|\nabla \phi \right|}^{2}\right)$$

*and G_c_ stands for the critical energy release rate (fracture toughness), which was considered the material property in* [24,25]*. In that case, it follows that:*

(5) $$\psi^{c}=\frac{3}{8\sqrt{2}}\frac{G_{c}}{l}$$

*However, note that according to the original threshold formulations proposed by Miehe et al. in* [39,40]*, in the case of a general elasto-plastic cyclic deformation process,* $\psi^{c}$ *should be considered the material fracture model parameter, to be defined either experimentally or empirically through numerical experiments. Therefore, it is not clear if the approach proposed in* [24] *is actually able to produce quantitatively meaningful results if an experimentally defined Gc were used to define* $\psi^{c}$ *according to Equation (5). On the contrary, it seems that in that case G_c_ should be treated as a purely numerical parameter, to be calculated from Equation (5) using* $\psi^{c}$ *defined from experimental stress–strain curves, making the approach given by (3)–(5) superfluous. Moreover, it can be shown that (5) is strictly valid only for brittle fracture. It corresponds to the definition* $\psi^{c}=0.5\;\sigma_{c}\varepsilon_{c}$*, with* $\sigma_{c}$ *and* $\varepsilon_{c}$ *being the critical (peak) stress and its corresponding strain, respectively, obtained by analytically solving a 1-D problem; see, e.g.,* [41].

Here the problem described in the Remark was avoided because we adopted a brittle fracture model, and $\psi^{c}$ has to be modified anyway in fatigue analyses due to the action of cyclic loading. Therefore, in the presented simulations, the initial value of $\psi^{c}$ is defined as in (5) using the experimentally estimated values for *G_c_*, identified with the procedures described in Section 2.1.1. The length scale parameter can then be calculated using the following expression:

(6) $$l=\frac{3}{4\sqrt{2}}\frac{G_{C}E}{{\left(\sigma^{c}\right)}^{2}}$$

where $\sigma^{c}$ corresponds to the ultimate tensile stress.

The degradation in fracture energy caused by fatigue is defined by a fatigue degradation function $\hat{F}\left(\bar{\psi }\right)$ as

(7) $$\psi^{c}\left(\bar{\psi }\right)=\frac{3}{8\sqrt{2}}\frac{G_{c}\left(\bar{\psi }\right)}{l},\;\;G_{c}\left(\bar{\psi }\right)=\hat{F}\left(\bar{\psi }\right)\;G_{c}$$

In this work, the logarithmic fatigue degradation function (reported in [24,25] in an erroneous form) is defined as

(8) $$\hat{F}\left(\bar{\psi }\right)={\left(1-\log\frac{{\bar{\psi }}_{\infty }}{\bar{\psi }}\right)}^{2},\;\;\;\mathrm{for}\;\bar{\psi }\in \left[{\bar{\psi }}_{\infty },10\;{\bar{\psi }}_{\infty }\right]$$

with ${\bar{\psi }}_{\infty }$ as the fatigue model parameter, which here is calibrated to capture the total life of the examined material given by an experimental S–N curve.

The governing equations in the strong form are the standard equilibrium equations with the corresponding natural and displacement boundary conditions, and the evolution equation of the phase-field, both obtained by adopting homogeneous natural boundary conditions for the phase-field (see [25,39] for the derivations). After introducing a few modifications, the evolution equation of the phase-field can be written as

(9) $$-l^{2}\Delta \phi +\left[1+H\right]\phi =H$$

Herein, H is the history field parameter introduced to impose the irreversibility condition of the phase field ($\dot{\phi }\left(t\right)\geq 0$), according to Miehe et al. [42]. Here it is defined as

(10) $$H\left(t\right):=\max_{\tau \in \left[0,t\right]}\;\;\tilde{D}\left(\psi_{e}\left(\tau \right)\right),$$

with

(11) $$\tilde{D}\left(\psi_{e}\left(\tau \right)\right)={\langle \frac{\psi_{e}\left(\tau \right)}{\psi^{c}\left(\bar{\psi }\left(\tau \right)\right)}-1\rangle }_{+}\;\;\mathrm{for}\;\;\;\forall \tau \in \left[0,t\right]$$

being the crack driving state function at some moment *τ* during the loading process. The Macaulay bracket was introduced in order to keep the phase-field bounded (*ϕ* ≥ 0).

The governing equations in the weak form are obtained by introducing definitions (7), (8), (10) and (11) in the internal weak form (1), and applying the principle of virtual work, as described in [24,25].

#### 2.2.2. Numerical Implementation

An interested reader can find important details about the discretization procedures for and numerical implementation of numerical models in the commercial program package Abaqus in [24,25,43]. An important modification to those works is a new cyclic skipping procedure developed in order to reduce computational time and memory demands.

Numerical PF models based on FEM are computationally demanding due to the requirement for dense mesh discretization around cracks, which requires a large computational time and memory resources. When solving HCF problems, these requirements are multiplied because of the large number of loading cycles that have to be simulated until reaching total failure. To reduce computational costs, herein a two-step cycle skipping technique is embedded into the existing numerical routines for the presented PF formulation, relying on the energy accumulation variable $\bar{\psi }$. The first cycle skipping step seeks the cycle in which the initial degradation of the bulk material occurs, satisfying the condition:

(12) $$\hat{F}\left(\bar{\psi }\right)\psi_{c}<\psi_{e}$$

as in [24]. Since prior to damage initiation a constant loading amplitude leads to a constant change in the strain energy density Δψ inside each cycle, the accumulated energy variable value can be calculated as

(13) $$\bar{\psi }=N_{1}\Delta \psi$$

By inserting (8) and (13) into (12), the number of cycles to crack initiation, which can be skipped after the first step, is computed via

(14) $$N_{1}=\frac{\bar{\psi }}{\Delta \psi }10^{-\sqrt{\frac{\psi }{\psi_{c}}}}$$

After the onset of damage, further cycle skipping is performed as described below. It is based on the extrapolation procedure proposed in [44]. For two consecutive loading cycles exhibiting changes in the accumulation variables $\Delta {\bar{\psi }}_{1}$ and $\Delta {\bar{\psi }}_{2}$, respectively, the number of cycles skipped is computed using the relation

(15) $$\Delta N_{2}=q\frac{\Delta {\bar{\psi }}_{2}}{\Delta {\bar{\psi }}_{2}-\Delta {\bar{\psi }}_{1}}$$

where q represents a fidelity parameter, which controls the maximum allowed number of skipped cycles. After finding the appropriate number of skipped cycles according to Equation (15), the result is used for extrapolation of the accumulation variable from the previously achieved value after N cycles as

(16) $$\bar{\psi }\left(N+\Delta N_{2}\right)=\bar{\psi }\left(N\right)+\Delta {\bar{\psi }}_{2}\Delta N_{2}+\frac{1}{2}\left(\Delta {\bar{\psi }}_{2}-\Delta {\bar{\psi }}_{1}\right){\left(\Delta N_{2}\right)}^{2}$$

The presented procedure is embedded into the FE software Abaqus by means of the subroutine UEXTERNALDB.

### 2.3. Numerical Modelling of Microstructure

**Figure 9:**
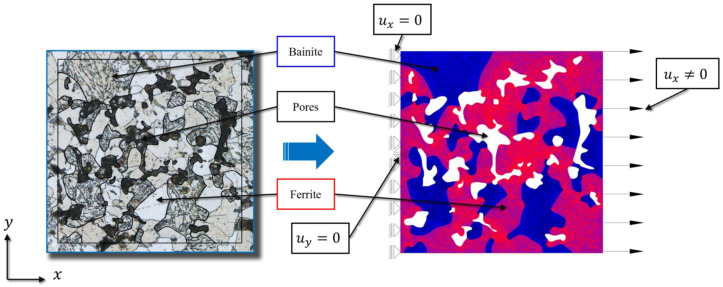
Metallography image of microstructure and extraction of 2D numerical model with corresponding boundary conditions.

Two-dimensional (2D) models of heterogeneous microstructures were extracted from high-resolution 4K metallography images, the specimens previously being polished and etched for a few seconds in 3% Nital fluid. Figure 9 shows the microstructure of the 6.5 g/cm^3^ sample and the process of extracting a corresponding numerical model. The models were chosen in a such way that the ferrite percentage is around 30% [32] for all densities. To acknowledge the fact that the aim of this contribution is studying fracture processes at the material microlevel, in this work the considered parts of the microstructure are called the Microscopic Volume Elements (MVE), rather than the Representative Volume Elements (RVE).

Two-dimensional (2D) MVEs were created, where the geometrical models of the microstructures were directly extracted from metallographic photographs using AutoCAD software. The considered microstructures were modelled to a high degree of accuracy, but the grain and pore boundaries were approximated by spline curves. The thus obtained graphical models were then exported into Abaqus software [45], where adequate meshes were generated. Since the pores and different phases are described with spline lines, the discretization process was not dependent on the resolution of lines, which would be the case if polylines were used.

Three different sizes of MVEs with side lengths of 0.2, 0.3 and 0.4 mm were modelled for each density in order to investigate size effects. The boundary conditions corresponding to the uniaxial tensile test (UTT) were prescribed on the MVE models by imposing displacements on the left and right vertical edges, while considering the remaining outer edges to be free surfaces (Figure 9). In all models non-zero horizontal displacements were imposed along the right edge, which correspond to the loading and vary during the loading process. Along the left edge, the horizontal degree of freedom (x axis) was suppressed. Moreover, at the node in the middle of the left edge, the vertical degree of freedom (y axis) was also suppressed in order to avoid rigid body motion. Homogeneous zero natural boundary conditions were defined along the upper and lower horizontal edges.

**Figure 10:**
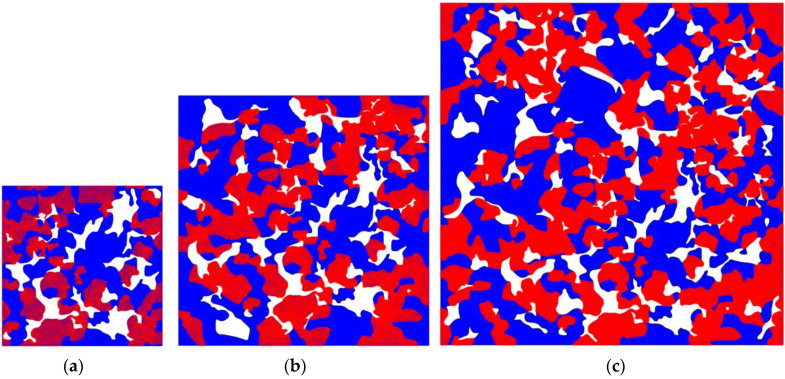
Different MVE size models for 6.5 g/cm^3^ density. The red region corresponds to the bainite phase, while the blue region corresponds to the ferrite phase. (**a**) 0.2 mm—Model S, (**b**) 0.3 mm—Model M, (**c**) 0.4—Model L.

The models presented were discretized using approximately 30,000, 70,000 and 130,000 quadrilateral finite elements for the 0.2 (Model S), 0.3 (Model M) and 0.4 (Model L) mm models, respectively, for all three densities (Figure 10). The average element size was h = 1.5 µm, chosen so that the microstructural topology could be captured in detail. The size of the elements is therefore much smaller than the size of individual grains and pores. Ferrite grains have the approximate size of 30 µm and the approximate size of bainite grains is 70 µm, while the pores range from 20–80 µm [33]. At the same time, the demand that h<1/2l, necessary to accurately resolve the phase field using the applied PF formulation, is satisfied. Numerical simulations were conducted on a workstation with an AMD CPU with 3.80 GHz base clock, 128 GB of RAM and acceleration with a NVidia RTX™ GPU card. The duration of each simulation was around 20, 30 and 54 h for the S, M and L models, respectively.

The average strains and stresses for the numerically obtained stress–strain diagrams presented in the following chapter are calculated for each MVE as:

(17) $$\sigma =\frac{RF}{L},\;\;\;\varepsilon =\frac{u}{L}$$

where *RF*, *u*, and *L* are the total reactive force, average horizontal displacement at the loaded side, and side length of MVE, respectively.

As presented in the introduction, various sophisticated PF models for microfracture in heterogeneous materials have been proposed, but they demand significant computational costs, and this problem would only be further exacerbated in HCF simulations with complex geometries, such as those encountered in the microstructures of sintered steels. Therefore, the following simplifications are adopted here:

- The interfaces between individual material phases are not modelled as separate entities or properties. Consequently, the present model is unable to simulate interphase fracture.
- Both material phases are considered to be elastically isotropic, with properties given in Table 3, with bainite regarded as being homogenous.
- The isotropic fracture surface energy defined in Equation (1) is adopted.
- Plastic deformation, which can normally be expected during the microfracture of sintered metals, is neglected.

From the above, it can be concluded that this model can be called a simplified PF model for HCF in multiphase sintered materials.

The present model is not suitable for accurate modelling of microfracture patterns in polycrystals, as anisotropy is not taken into account. However, in this work we observed our heterogeneous material at a higher length scale, where the material phases are homogenized and are not considered polycrystals.

## 3. Results and Discussion

### 3.1. Quasi-Static Fracture

**Figure 11:**
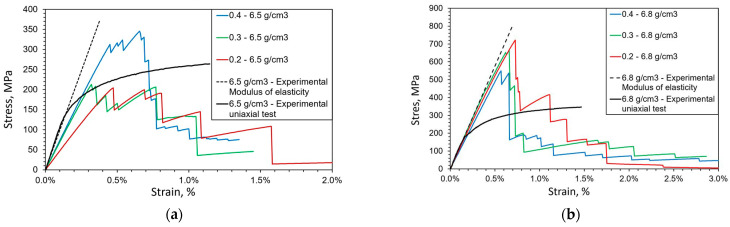

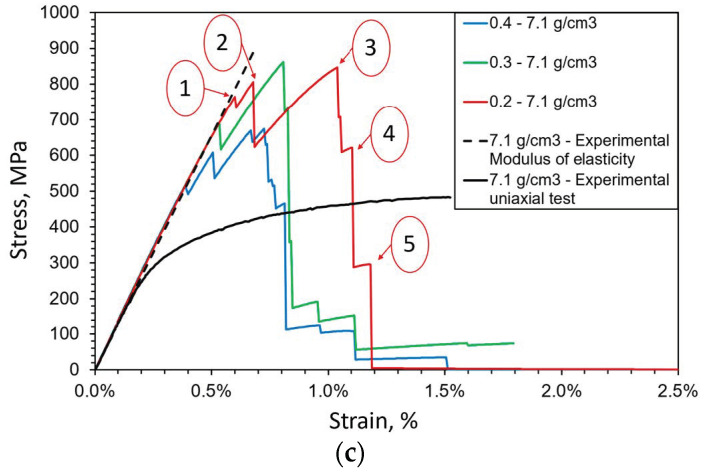
Average stress–strain response with respect to size in numerical models of microstructure for different densities: (**a**) 6.5 g/cm^3^, (**b**) 6.8 g/cm^3^, (**c**) 7.1 g/cm^3^. The points marked by numbers from 1 to 5 in Figure (**c**) are used to present the evolution of microcracks in Figure 12.

First, only the elastic response from the numerical model of the microstructure was investigated. A comparison of the experimentally obtained macroscopic modulus of elasticity and the effective modulus of elasticity obtained using the numerical model is presented in Figure 11. The simulation results for the specimen with a density of 6.5 g/cm^3^ exhibit a larger scattering of results and higher error than the results for the other two densities, indicating a more pronounced size effect. In addition, for this density the value of the effective elastic modulus increases with increasing MVE size, which is not a physical behavior. This indicates that, for this density, larger MVEs should be considered to establish a representative volume element (RVE) for elastic behavior.

Important to note here is that, for smaller porosity levels, the bulk (macro) modulus of elasticity can be predicted quite accurately using these numerical models for a heterogeneous microstructure, with the local elastic material parameters (the moduli of elasticity of different metallic phases) obtained using the applied instrumented micro-indentation.

Microcrack initiation and propagation were analyzed next. From the average stress–strain responses presented in Figure 11 it is obvious that the present model is unable to accurately capture the global nonlinear response, mainly because the plastic deformation of individual material phases is neglected in the numerical model. Due to the absence of plastic deformation in the numerical models, the final elongation is generally grossly underestimated, and overly early advent of sudden fracture (unstable global crack propagation) through the material is predicted. However, the size effect is still visible, in accordance with the observations in [14]. The modelled MVEs (except for the S model with 6.5 g/cm^3^) predict a higher peak stress (the tensile strength) than the macroscopic sample, which is again consistent with size effect phenomena. Sudden vertical drops in stress values are visible in post-peak (softening) responses, which are caused by the brittle fractures of small “bridges” between the pores (particle necks appearing during the sintering process as bonds formed between powder particles). A small bridge breaks when a microfracture initiated at a pore at one end of the bridge propagates until reaching a pore positioned at another side of the bridge. In addition, note that the numerical model correctly predicts that tensile strength increases with a reducing porosity level in the material, which is clearly visible at the macrolevel [33]. This could be attributed to greater distances between the initiated microcracks in the material with higher density, i.e., lower porosity. In that case, higher loading is needed to cause growth and coalescence of microcracks sufficient to trigger global softening.

**Figure 12:**
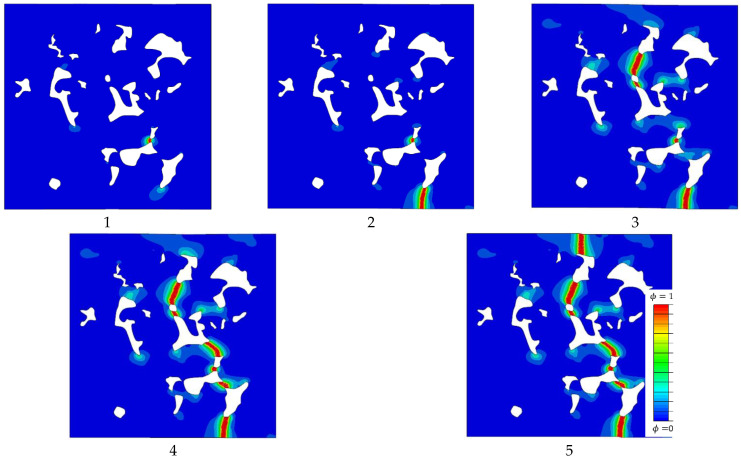
Microcrack propagation in S model (size 0.2 × 0.2 mm) with 7.1 g/cm^3^. 1–5 show phase field distribution in the model for the loading states that correspond to the points marked 1–5 on the stress–strain curve in Figure 11c.

The predicted fracture patterns were examined next. The propagation of microcracks during the loading process is illustrated in Figure 12. The areas with high values of the phase field correspond to highly damaged materials. Theoretically, the points where ϕ=1 define well-formed microcracks, i.e., crack paths. On the other hand, in areas where the phase field is equal to zero, the material is intact. For points where the phase field is in between these extreme values, the material stiffness and fracture properties, i.e., the modulus of elasticity and fracture toughness, degrade due to sustained damage. The cracks propagate from one pore to another until they coalesce into larger cracks, which finally results in a macroscopic fracture.

**Figure 13:**
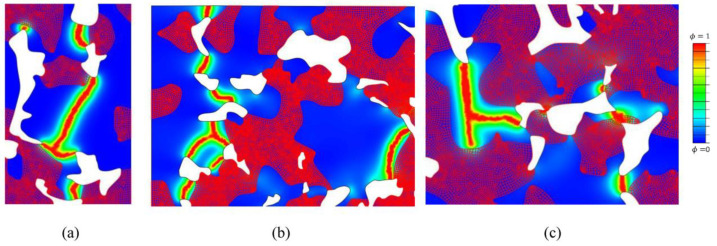
Transgranular cracks predicted using the PF model. Blue areas correspond to bainite and red to ferrite. (**a**) detail of L model of 6.8 g/cm^3^, (**b**) detail of M model of 6.8 g/cm^3^, (**c**) detail of M model of 7.1 g/cm^3^.

A closer inspection of crack patterns reveals that, in accordance with expectations, the model predicts transgranular microcracks, where cracks pass through different phases (ferrite or bainite). In Figure 13 it is visible that in most cases the microcracks propagate through a bainite zone, since bainite has stiffer behavior (higher modulus of elasticity) and lower fracture resistance (lower fracture toughness) than ferrite. Cracks typically initiate in bainite zones at the edge of pores, which act as stress concentrators. The further propagation of a microcrack is driven by the microstructure’s topology, because the relative distribution of material phases and pores in the microstructure dictates the stress distribution and fracture energy.

An interesting phenomenon where a ferrite phase stops the further propagation of a crack may be clearly observed in Figure 13c. This indicates that it could be necessary to include intergranular crack propagation into the model if realistic crack patterns are to be captured using the proposed strategy. Exceptionally, if the minimum strain energy is found for crack propagation through a ferrite zone, the crack continues to propagate through the ferrite zone. This can occur if the ferrite zone is located along a small “bridge” between two pores (in Figure 13c some small “bridges” pass through ferrite). The microcracks often merge or branch, as is visible in Figure 13a–c.

From the presented results it can be concluded that the presented model is qualitatively able to capture the propagation of microcracks by the mechanism of collapsing “bridges” between pores, also visible in the SEM images (Figure 8).

### 3.2. Cyclic Loading

**Figure 14:**
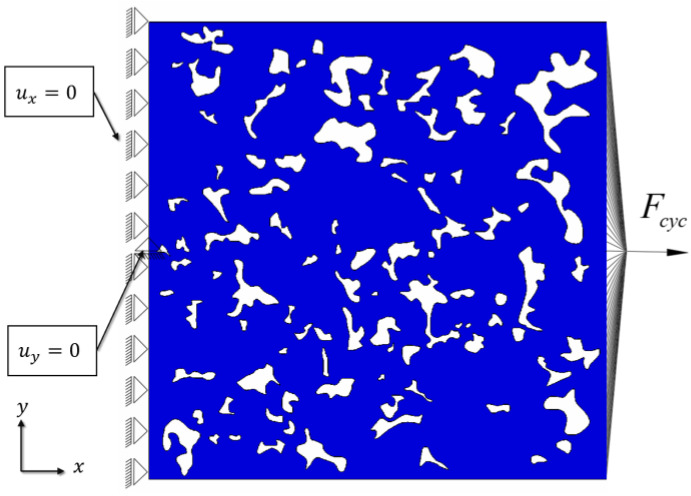
Boundary conditions of the 6.8 g/cm^3^ L model for fatigue modelling.

The PF model for fatigue failure, employing the degrading fracture energy defined by (7) and the cycle skipping technique for speeding up the calculations, was used for all fatigue simulations. Identical geometric models of the microstructure and finite element meshes as those for monotonous quasi-static fracture analysis were used. For the purpose of brevity, here only the results obtained from L-size microstructure models are presented. Instead of the loading used in the static case, herein loading conditions were imposed on the microstructure by means of kinematic restraints, where the concentrated nodal force $F_{cyc}$ is prescribed to a reference node coupled via the imposed kinematic constraints to all nodes positioned at the loaded edge (Figure 14). Moreover, the left edge was restrained in the *x* direction and the middle node on the left edge in the *y* direction.

The material parameters given in Table 1 and Table 2 were used in simulations, and the loading ratio for all densities was kept at *R* = 0.05, as in the experimental testing discussed in Section 2.1.2. The value of fatigue parameter ${\bar{\psi }}_{\infty }$ was chosen as 50 (for both phases) in order to best fit the experimental S–N curves.

Using the cycle skipping feature explained in Section 2.2, the duration of simulations was reduced from a few weeks (5–6) to a few (4–5) days. Another benefit in comparison to the formulation from [24] was a significant reduction in memory demands. For an HCF test with $N_{f}\approx 10^{7}$ the present algorithm does not require more than 150 GB (for a numerical model with more than 100 000 finite elements), which is available in most desktop computers nowadays. The authors believe that a similar HCF simulation without a cycle skipping feature would possibly require a few terabytes of data.

**Figure 15:**
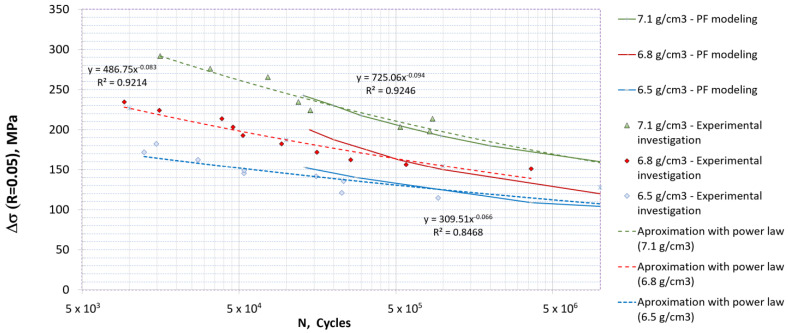
Wöhler curves obtained using the PF model for fatigue fracture for different densities of sintered steel. The L-size numerical model of the material microstructure is applied for all curves.

Numerically obtained Wöhler curves are presented Figure 15. The model predicted the fatigue total life relatively well in the HCF regime. For a smaller number of cycles it seems that the model tended to overestimate the experimentally determined total life, probably due to neglecting plastic deformation and intergranular cracks. This could be important because the local plastic flow could be significant, especially around small “bridges” between pores, and could contribute significantly to the formation of microcracks [3]. In addition, it seems that in the present model the ferrite phases sometimes stop the further propagation of microcracks, similarly to pores, while in reality the crack could propagate along the interface between the adjacent ferrite and bainite phases. On the other hand, a slight underestimation of the total life could be observed for low loading, possibly because stress concentrations in MVEs lead to large energy accumulation per cycle and therefore the energy accumulation variable $\bar{\psi }$ grows faster, resulting in the underestimation of critical fracture energy during the process.

As already explained, for accurate simulation of microfractures, elasto-plastic analysis would be a preferable approach, but characterizing the plastic parameters of individual material phases in multi-phase materials is by no means a simple task and requires appropriate nano-indenters. Even then, its accuracy depends greatly on the method applied for reconstructing stress–strain curves [27,29]. During our research, we were not able to obtain reliable data for plastic parameters of ferrite and bainite. In addition, in elasto-plastic PF models with a threshold, the critical fracture energy per volume (the specific fracture energy) and not the critical release energy release rate *G_c_* should figure as the model parameter, as explained in Section 2.2. This, however, demands that the maximum stress (i.e., the tension strength) be characterized for each phase, which is another challenge in instrumental indentation. Although plasticity is not included in the proposed model, it could nevertheless be said that the influence of plastic deformation is implicitly captured in the present modelling paradigm in HCF regime. It is also noted that in [24] a way to fine-tune the fatigue degradation function $\hat{F}\left(\bar{\psi }\right)$ is proposed, which, however, is not pursued in the scope of this work.

**Figure 16:**
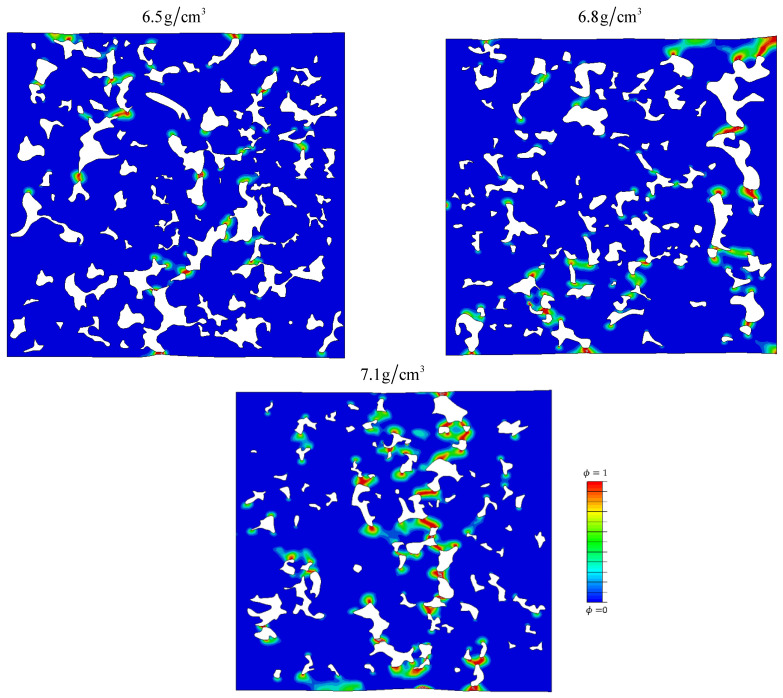
Numerically obtained microcrack patterns before the onset of total failure for identical loading cases with Δσ=152 MPa. Figures show the distribution of the phase field at the moment right before complete failure.

In Figure 16 the microcrack patterns in MVEs just before the onset of total failure are shown for the same loading case with Δσ = 152 MPa. For higher densities, the microcracks coalesce into larger cracks vertical to the loading direction (horizontal in images) in the “weakest” cross sections (those with the largest ratio of pores along the section). This behavior qualitatively corresponds well to the brittle fatigue fracture observed in macroscopic samples during UTT (see Figure 1c). In the sample with the highest level of porosity, it seems that large cracks are formed in a more “diffuse” manner by joining the cracks in the smallest “bridges” in the microstructure.

**Figure 17:**
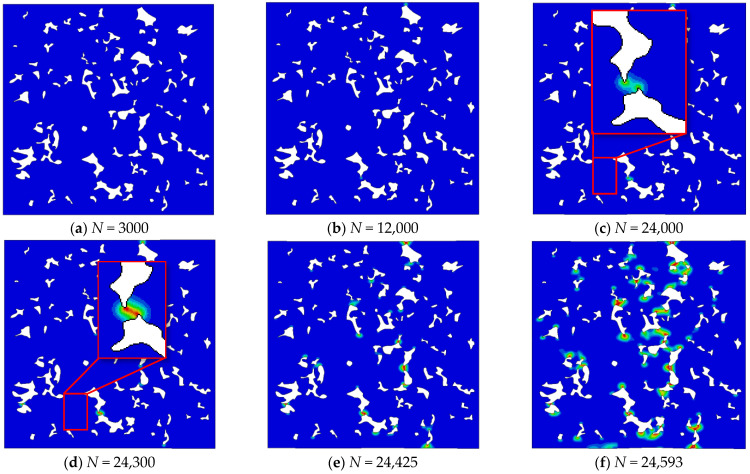
Evolution of fatigue microcracks for density 7.1 g/cm^3^ and Δσ=152 MPa. (**a**–**f**) show phase field distribution in the model after different numbers of loading cycles.

This is further demonstrated in Figure 17, where the evolution of microcracks in the microstructure with 7.1 g/cm^3^ density is presented. The first microcracks appear at certain small bridges (Figure 17c,d). Gradually, other bridges collapse (Figure 17e) with a tendency to connect and create one larger microcrack (Figure 17f). A similar observation was noticed by Falkowska and Seweryn [46] in their experimental SEM observation of sintered steel samples with different densities submitted to cyclic loading.

**Figure 18:**
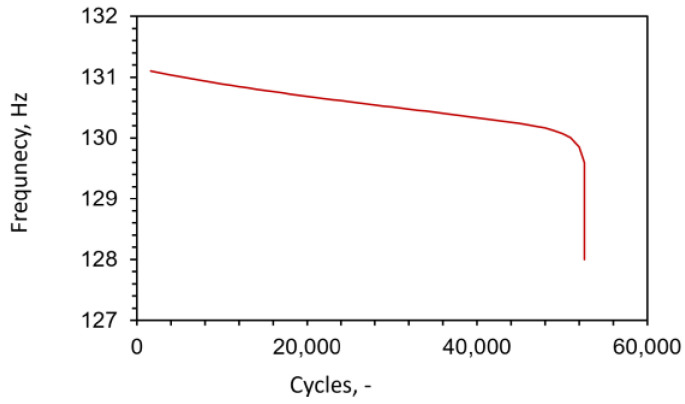
Resonant frequency with respect to number of loading cycles for a specimen with density 6.5 g/cm^3^.

In addition, the simulation results (see Figure 17) imply that most of the fatigue degradation takes place in the last few hundred cycles before complete failure. Such behavior is typical for the degradation function (Equation (8)) used in this paper, and is similar to the observations gained by inspecting loading–unloading cycles under static loading (Figure 6), which also suggest that the fracture appears suddenly after the material suffers a certain amount of degradation. To confirm this result, measurement of resonant frequency during experimental testing was performed, since any deviation in frequency can be continuously monitored and directly coupled to microcrack propagation inside the tested specimen (while maintaining a fixed room temperature). Such a change in the resonant frequency is displayed in Figure 18, where a sudden drop is visible just a few hundred cycles before the final failure.

## 4. Conclusions

A fast numerical method based on a phase-field model for fracture is proposed for estimating the fatigue total life of multiphase sintered steels through simulations of fatigue microfracture in the material. In contrast to existing models for multiphase materials, herein a simplified numerical phase-field model for fatigue fracture based on the Finite Element Method is employed in order to reduce the large computational resources needed for numerical fatigue simulations. Plastic deformation is neglected in the present model, the influence of material interfaces is not modelled, and the material phases are assumed to be isotropic. A new cycle-skipping algorithm is also proposed, which can yield further savings in computation costs. The model can be calibrated by identifying microscopic elastic material parameters using instrumented indentation, while fracture parameters are calibrated from experimental S–N curves.

The model exhibits some size effects, such as reducing the effective maximum stress and elastic modulus of sintered steel with increasing sizes of considered microscopic volume elements (MVEs). The results indicate that the model captures well the initiation of the first damage in the microstructure, where microcracks first appear in the narrowest bridges between two closely located pores. In addition, the initiation and propagation of larger fatigue cracks in the material, corresponding to the brittle fracture observed experimentally in the examined material, are qualitatively captured. This confirms the experimentally confirmed fact that fracture propagation in porous sintered steels is strongly driven by porosity. However, the proposed model is unable to yield realistic crack patterns in the microstructure due to the assumed simplifications. Nevertheless, the obtained results indicate that the proposed approach is suitable for investigating the influence of porosity on the fatigue total life in a high cycle fatigue (HCF) regime. Therefore, it could be used for preliminary investigation in research dealing with the influence of microstructure on fatigue failure in similar sintered steels.

In order to obtain more accurate predictions for fracture patterns, future research will be dedicated to extending the model to ductile failure by including plastic deformation, as well as including models of intergranular crack propagation. Thereby, special attention should be paid to the mechanical characterization of local material plastic and fracture parameters. Besides improvements in constitutive modelling of the microstructure, an extension towards 3D modelling will be also considered. Based on the results obtained for fatigue response at the material microscale, a procedure will be proposed for the assessment of macroscopic fatigue parameters based on explicit numerical modelling of material fatigue at the microscale.

## Figures and Tables

**Table 1:** Microstructural properties according to metallographic imaging and instrumented indentation [33]. V_0_ is the volume of bulk material without pores (material matrix), V_p_ is the total volume of the pores.

Sintered Density, g/cm^3^	Phase Volume Fraction, %			Microproperties	
	Porosity, k_p_ = V_p_/V_0_	Ferrite, k_f_ = V_f_/V_0_	Bainite, k_b_ = 1 − k_f_	Ferrite	Bainite
				Modulus of Elasticity, GPa	
6.5	20	33 ±2.5	xxx	185.3 ± 29.5	238 ± 29.6
6.8	14.7	30 ±3.7	xxx	189.1 ± 22.8	240 ± 17.2
7.1	9,9	31 ± 4.1	xxx	183.6 ± 20.7	238 ± 22.4

**Table 2:** Fracture toughness of microstructural constituents obtained via multicyclic spherical indentation.

	Fracture Toughness (Energy Release Rate), *G*_C_, N/mm
bainite	8.45
ferrite	14.5

**Table 3:** Identified elastic properties [33] and fracture toughness of material phases.

	Bainite	Ferrite
Modulus of elasticity, *E*, MPa	239,000	186,000
Poisson ratio, *v*, -	0.28	
Energy release rate (Fracture toughness), *G_C_*, N/mm	8.5	14.5

## Data Availability

Data sharing not applicable.
